# Abnormalities of synaptic mitochondria in autism spectrum disorder and related neurodevelopmental disorders

**DOI:** 10.1007/s00109-020-02018-2

**Published:** 2020-12-18

**Authors:** Liliana Rojas-Charry, Leonardo Nardi, Axel Methner, Michael J. Schmeisser

**Affiliations:** 1 Institute for Microscopic Anatomy and Neurobiology, University Medical Center of the Johannes Gutenberg-University, Duesbergweg 6, 55128 Mainz, Germany; 2 Institute for Molecular Medicine, University Medical Center of the Johannes Gutenberg-University, Langenbeckstraße 1, 55131 Mainz, Germany; 3 Focus Program Translational Neurosciences (FTN), University Medical Center of the Johannes Gutenberg-University, Mainz, Germany

**Keywords:** Autism spectrum disorder, ASD, Synapse, Mitochondria, Neurodevelopmental disorders

## Abstract

Autism spectrum disorder (ASD) is a neurodevelopmental condition primarily characterized by an impairment of social interaction combined with the occurrence of repetitive behaviors. ASD starts in childhood and prevails across the lifespan. The variability of its clinical presentation renders early diagnosis difficult. Mutations in synaptic genes and alterations of mitochondrial functions are considered important underlying pathogenic factors, but it is obvious that we are far from a comprehensive understanding of ASD pathophysiology. At the synapse, mitochondria perform diverse functions, which are clearly not limited to their classical role as energy providers. Here, we review the current knowledge about mitochondria at the synapse and summarize the mitochondrial disturbances found in mouse models of ASD and other ASD-related neurodevelopmental disorders, like DiGeorge syndrome, Rett syndrome, Tuberous sclerosis complex, and Down syndrome.

## Introduction

Autism spectrum disorder (ASD) is a neurodevelopmental condition that starts in childhood and prevails across the lifespan, symptoms are variable, and a substantial increase in ASD diagnosis has been reported during the last 40 years [1]. A significant part of ASD cases is associated with mutations in synaptic proteins, suggesting an impairment of synaptic transmission as a primary underlying cause [2–7]. Synaptic activity is an energetically expensive process that consumes a large proportion of the adenosine triphosphate (ATP) generated in neurons, which is mainly produced by mitochondria through oxidative phosphorylation (OXPHOS) [8]. Mitochondria are present in approximately half of all presynaptic boutons, and synapses that contain mitochondria have more vesicles [9]. Postsynaptic mitochondria are less abundant and have a more tubular form than presynaptic mitochondria [10], indicating that distinct morphological changes in dendrites and axons occur to adjust their shape to energetic or other needs [11]. In addition, local synthesis of new proteins occurs in axons and dendrites and depends on mitochondria that provide energy during synaptic plasticity [12]. Besides their role as energy providers, mitochondria also act as calcium (Ca^2+^) buffers that shape the synaptic response [13]. Hence, their presence at the synapse serves not only to produce ATP but also to control local Ca^2+^ concentrations ([Ca^2+^]) and neurotransmitter release, which is essentially triggered by a sudden increase in Ca^2+^ concentration. The synaptic [Ca^2+^] is tightly regulated by efflux through the plasma membrane and uptake into the spine apparatus, a sub-compartment of the smooth endoplasmic reticulum (sER), and mitochondria [14–17]. Genetically encoded Ca^2+^ sensors have provided evidence that individual hippocampal and cortical synapses with mitochondria accumulate less synaptic Ca^2+^ than those lacking these organelles [18, 19]. Based on the importance of synaptic signaling in ASD and the relevance of mitochondria in synaptic activity, we here aim to summarize the current knowledge about the role of synaptic mitochondria in ASD and other ASD-related neurodevelopmental disorders.

## Functions of neuronal and synaptic mitochondria

The brain consumes large amounts of oxygen—20% of the whole body’s consumption—and most of this oxygen is used to generate ATP through OXPHOS in mitochondria [8]. Mitochondria provide 93% of the ATP that the brain demands [20]. This ATP is used to support synaptic transmission, a very energy-demanding process. ATP is necessary to power ion pumps, support ion gradients, and maintain vesicle recycling and mitochondrial movement. Remarkably, the number of mitochondria in synaptic terminals and axons exceeds the predicted energy needs [20], which implies that they have additional functions at the synapse, like the buffering of intra-spine Ca^2+^ levels that directly influence the firing probability of neurons [14, 21].

The function of mitochondria as Ca^2+^ buffers and ATP producers also relies on the ER [22], which stores the highest concentrations of Ca^2+^ ions. The points of contact between the ER membrane and mitochondria are called mitochondria-ER contact sites (MERCs) or mitochondria-associated membranes (MAMs) [23]. MAMs are crucial for controlling Ca^2+^ concentrations in neurons through the ER channels inositol 1,4,5-trisphosphate receptors (IP3R), the sarcoendoplasmic-reticulum Ca^2+^ ATPase (SERCA), the glucose-regulated protein 75 (Grp75), the voltage-dependent anion channel (VDAC1), and the mitochondrial Ca^2+^ uniporter (MCU) [24]. Another Ca^2+^ modulator, the receptor chaperone Sigma 1 (S1R), localizes in MAMs in a complex with type 3 IP3R [25]. Other tethering complexes are formed by proteins like the protein tyrosine phosphatase-interacting protein 51 (PTPIP51), the vesicle-associated membrane protein-associated protein B (VAPB), and the B cell receptor–associated protein (BAP31), which are also associated with Ca^2+^ handling [26]. Interestingly, mutations in VAPB and S1R are related to neurodegenerative diseases [27, 28]. MAMs are frequent in neurons, and besides the regulation of Ca^2+^ signaling, they are involved in synaptic transmission, since the absence of VAPB-PTPIP51 in synapses leads to a reduced number of dendritic spines and decreased synaptic activity [29, 30].

**Fig. 1:**
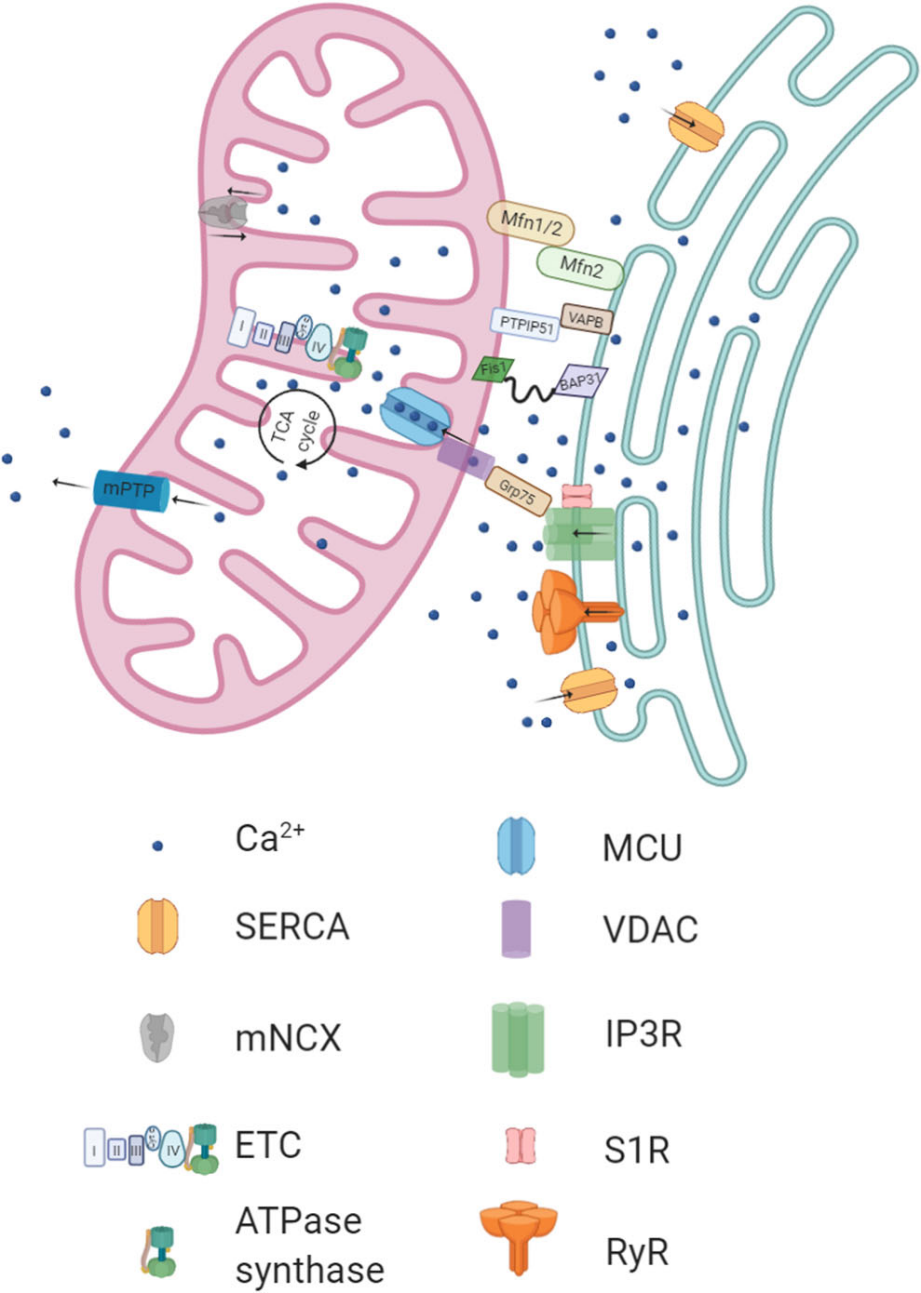
Mitochondrial-ER contact sites in neurons. The endoplasmic reticulum (ER) and mitochondria are connected by several proteins depicted here. Mitofusin 2 (Mfn2) regulates the tethering between the two organelles by a direct interaction through its heptad repeat domains (HR)[40]. Mitofusin 1 (Mfn1) participates in this process and remains in the mitochondria. Other proteins like tyrosine phosphatase-interacting protein 51 (PTPIP51) and the vesicle-associated membrane protein-associated protein B (VAPB) also build bridges to connect both organelles [26]. The mitochondrial fission protein (Fis1) forms a complex with the B cell receptor–associated protein (BAP31) that signals for apoptosis [41]. The sarcoendoplasmic-reticulum Ca^2+^ ATPase (SERCA) transports Ca^2+^ into the ER. The Ca^2+^ release channel, inositol 1,4,5-trisphosphate receptor (IP3R), is composed of 4 subunits [42] and interacts with a Ca^2+^ modulator, the receptor chaperone Sigma 1 (S1R). The ryanodine receptor (RyR) is another Ca^2+^ release channel. Ca^2+^ ions enter the mitochondrial matrix through the mitochondrial calcium uniporter (MCU) and activate enzymes that stimulate the tricarboxylic acid (TCA) cycle and the electron transport chain (ETC) [31–33]. Excessive Ca^2+^ ions are released into the cytosol through the mitochondrial permeability transition pore (mPTP) and the Na^+^/Ca^2+^ exchanger (mNCX) [37, 38]. Arrows indicate the direction of Ca^2+^ flow. Created with BioRender.com

Cytosolic Ca^2+^ ions that enter the mitochondrial matrix through the MCU increase OXPHOS by stimulating pyruvate dehydrogenase phosphatase rendering the pyruvate dehydrogenase complex more active [31]. Ca^2+^ also activates other citrate cycle enzymes, like isocitrate dehydrogenase and alpha-ketoglutarate dehydrogenase [32, 33]. Besides MCU, additional mitochondrial uptake mechanisms have been identified in heart and liver cells, like the mitochondrial ryanodine receptor (mRyR) and the rapid mode of uptake (RaM) [34–36]. Ca^2+^ release into the cytosol is executed through either the mitochondrial permeability transition pore (mPTP) or the Na^+^/Ca^2+^ exchanger (mNCX) [37, 38]. The increase in cytosolic Ca^2+^ triggers synaptic vesicle exocytosis and, consequently, neurotransmitter release. Mitochondrial Ca^2+^ uptake inhibition contributes to increased cytosolic Ca^2+^, which causes depletion of synaptic vesicles [39]. See Fig. 1 for a representation of the MAM proteins and their role in ER-mitochondrial Ca^2+^ transfer.

Mitochondria are not entirely uniform in individual cells; they vary in size, morphology, and metabolic activity [43]. In axons, mitochondria are mostly round and not so numerous compared with perinuclear mitochondria, which form complicated networks. In general, mitochondria are dynamic organelles that adapt their morphology and distribution in neurons to fulfill specific needs [44, 45]. The constant shape change of mitochondria is called mitochondrial dynamics. It involves fusion and fission processes performed by a set of specific mitochondrial proteins involved in several diseases [46–48]. Mitochondria fuse to form networks, repair their damage, and share genetic information [49]. Mitochondrial fusion is accomplished by the GTPases Mitofusin 1 (Mfn1) and Mitofusin 2 (Mfn2), localized in the outer mitochondrial membrane (OMM). The process occurs through their hydrophobic domains, essential for association with other Mfns in proximal mitochondria [40, 50]. Another protein involved in this process is the dynamin-like 120 kDa protein or optic atrophy protein 1 (Opa1), which is anchored in the inner mitochondrial membrane (IMM) and necessary for the formation of cristae [51]. The conditional knockout of Mfn2 causes neurodegeneration in the hippocampus and cortex and increased oxidative stress [52]. Mfns also form bridges between the ER and mitochondria [53], and the mutation of *MFN2* is associated with hereditary Charcot-Marie-Tooth disease [54, 55].

Another dynein-like GTPase, the dynein-related protein 1 (Drp1), controls mitochondrial fission in neurons. Drp1 is found in the cytosol, oligomerizes, and is transported to mitochondria to constrain membranes by protein adaptors like the mitochondrial fission protein (Fis1), the mitochondrial fission factor (Mff), the mitochondrial dynamics protein 51 (MiD51/MIEF1), and the mitochondrial dynamics protein 49 (MiD49/MIEF2) [56]. Drp1 is the subject of several post-translational modifications that impact its activity. In neurons, phosphorylation of Drp1 at Ser616 by cyclin-dependent kinase 1 (CDK1) stimulates mitochondrial fragmentation. Meanwhile, phosphorylation at Ser637 by protein kinase A (PKA) and Ca^2+^/calmodulin-dependent protein kinase Iα (CaMKIα) inhibits mitochondrial fission [57, 58]. Modifications like nitrosylation promote Drp1 phosphorylation at Ser616, and consequently, mitochondrial fission in primary hippocampal neurons [59, 60]. Mitochondrial fusion and fission need to be balanced for neuronal health [61]. The deletion of Drp1 in adult mouse forebrain impairs spatial working memory and synaptic function [62, 63]. Reduced oxygen consumption and ATP concentrations are also found in isolated mitochondria from the hippocampal region.

The postsynaptic compartment is critical for triggering long-term potentiation (LTP), which underlies learning and memory formation. A recent study provides evidence about the interplay between mitochondrial fission and LTP [64]. The expression of a dominant-negative mutant of Drp1 that prevents mitochondrial fission in primary hippocampal neurons impairs LTP in cultured cells and hippocampal slices. Nevertheless, this study was performed in vitro. The relationship between LTP and mitochondrial dynamics is still unknown for in vivo models. It is also unclear whether mitochondrial morphology regulation solely depends on the increase of cytosolic [Ca^2+^], which takes place as the triggering step for synaptic signaling. The Wnt/Ca^2+^ signaling pathway is considered a regulator of mitochondrial dynamics at the postsynapse. The stimulation of hippocampal CA1 slices with the ligand Wnt-5a influences the morphology and the number of mitochondria in this compartment as measured by transmission electron microscopy, probably through the phosphorylation of Drp1, which was found augmented at Ser-616 and decreased at Ser-637, leading to mitochondrial fission [65]. A study from 2018 showed that neurons devoid of Mff release less presynaptic vesicles, providing further evidence of the essential role of mitochondrial morphology in synaptic activity [66].

Fragmented mitochondria are also related to mitophagy, a particular mechanism to remove unhealthy mitochondria observed in the soma and distal axons [67–69]. Damaged mitochondria are eliminated in a process regulated by the PTEN-induced putative kinase 1 (PINK1) and Parkin, a cytosolic E3 ubiquitin ligase. Under mitochondrial stress conditions, PINK1 remains in the OMM and phosphorylates ubiquitin on Ser65, which translocates Parkin to the mitochondria and initiates the degradation of the defect organelles through autophagy [70, 71]. The removal of dysfunctional mitochondria is required for preserving neuronal homeostasis [72, 73]. The PINK-Parkin pathway is the most-studied mechanism because mutations in these proteins are linked to the familial forms of Parkinson’s disease [74]. However, in vivo studies demonstrated that mitophagy also occurs in the absence of Pink1 in different populations of dopaminergic neurons, concluding that other mitophagy pathways exist [75]. This study also presented evidence that the expression of Pink1 differs according to brain region, being most extensively expressed in the striatum, neocortex, cerebellum, and spinal cord.

Mitochondria are also actively transported within axons to reach distant parts according to the energy requirements, mediated by AMPK signaling [76]. There is a direct correlation between synaptic activity and the transport and distribution of mitochondria [77]. During synaptic activity, mitochondria remain at presynaptic terminals and postsynaptic dendritic spines [78]. The transport of mitochondria is performed by a specialized machinery that works together with the cytoskeleton. It has been studied mainly in neurons due to the long distances these organelles travel to reach their destinations. The anterograde transport is mediated by Kinesin-1, which attaches to mitochondria through anchoring proteins like the Ca^2+^-sensing protein mitochondrial Rho GTPase 1 (Miro1), the microtubule-based motors dynein-dynactin, and the motor adaptors trafficking kinesin protein 1 and 2 (TRAK1 and TRAK2) [79, 80]. The retrograde movement is less well characterized but driven by the motor adaptor TRAK2 associated with dynein, which constitutes the essential retrograde mitochondrial motor [81]. Mitochondrial axonal transport during synaptic activity is also regulated by syntaphilin (SNPH), a protein that binds to Kinesin-5 to immobilize mitochondria. Presumably, SNPH anchors mitochondria to the microtubules [82]. One study performed in primary cortical neurons demonstrated that SNPH facilitates the removal of damaged mitochondria in axons by forming vesicles with late-endosomes, independently of the mitophagy system [83]. Interestingly, a recent study performed in ganglion cell dendrites of the intact retina challenged the accepted view of mitochondria as highly mobile organelles [84]. Faits et al. found that dendritic mitochondria remain stationary in mature neurons even when stimulated with Ca^2+^ transients, showing the complexity of mitochondrial positioning in dendrites and the differences found in cultured cells and whole tissues.

Most studies on mitochondrial function at synapses have focused on the presynaptic specialization, while the role of postsynaptic mitochondria is less explored. The postsynaptic density (PSD) is a highly specialized compartment that contains hundreds of different proteins that constantly change composition according to synaptic activity. Mitochondria are mainly seen in the dendritic shafts, but they can also reach spines [85]. Studies performed in hippocampal neurons demonstrate that reducing the mitochondrial content in dendrites decreases the number of spines and synapses [78].

**Fig. 2:**
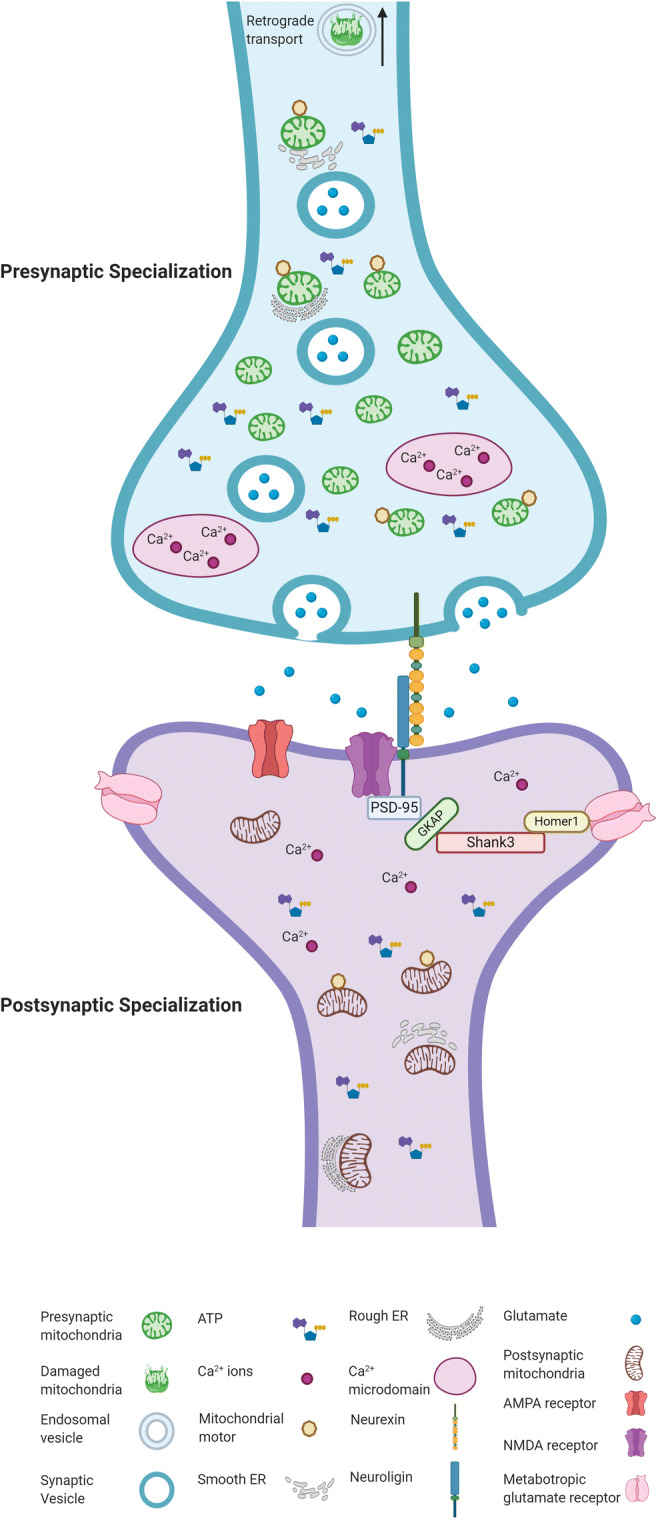
Synapse structure and mitochondria. The figure represents some of the recognized mitochondrial functions in a glutamatergic synapse. Presynaptic mitochondria are smaller and more abundant than postsynaptic mitochondria in the hippocampus [86]. Postsynaptic mitochondria have dense cristae, and tubular shapes, and make contacts with the ER [10, 87]. Mitochondrial movement increases in axons and is greater than in dendrites, although some mitochondria remain stationary (represented by the absence of mitochondrial motors) [88]. Not all of the presynaptic boutons contain mitochondria (only less than half), but the synapses with mitochondria have more vesicles and are associated with increased synaptic activity [9, 89]. Augmented Ca^2+^ concentrations are detected in microdomains (depicted in ovals) [90]. Neurexin, Neuroligin, and SHANK are synaptic scaffolding proteins implicated in ASD [91]. Abbreviations: PSD-95 (postsynaptic density protein 95); GKAP (guanylate kinase-associated protein). Created with BioRender.com

Although the current understanding of the role of mitochondria at the synapse has considerably increased (see Fig. 2 for a summary), many essential questions remain unanswered. One is how synapses without mitochondria handle energy requirements. Another one is whether and how distinct neuronal populations differ in mitochondrial shape and function since most studies have been performed in hippocampal and cortical neurons.

## Mitochondrial dysfunction in ASD

### ASD caused by mutations and loss of function of SH3 and multiple ankyrin repeat domains (SHANK) proteins

Several neurodevelopmental disorders are characterized by a combination of metabolic disease and synaptic disturbances [92, 93]. SHANK proteins, for example, predominantly serve as adaptor and scaffolding proteins in the PSD and connect ionotropic and metabotropic glutamate receptors (mGluR) with the actin cytoskeleton [94]; their mutations are implicated in multiple neuropsychiatric disorders, including ASD, schizophrenia, and intellectual disability to name the most frequently occurring [95–99].

Proteomic analysis of Shank3 complexes isolated by immunoprecipitation from synaptosomal fractions found an interaction of Shank3 with several mitochondrial proteins [100]. In this study, synaptosomal fractions were treated with different concentrations of ZnCl_2_, which binds to the C-terminal alpha motif domain of Shank3 and promotes its scaffolding and stabilization at the PSD. Interestingly, several mitochondrial proteins were identified in the interactomes analyzed after Zn^2+^ induction, including core subunits of the mitochondrial membrane ATP synthase, the NADH dehydrogenase 1, the cytochrome c oxidase, and the ADP/ATP translocase 4, accounting for 22% of the proteins identified. This work was performed in Shank3-overexpressing mice.

Phelan McDermid syndrome (PMS), or 22q13.3 deletion syndrome, is characterized by different symptoms, including developmental delay, speech difficulties, and ASD-like behavior. This syndrome is caused by the loss of one functional copy of *SHANK3* [101]. Mitochondrial dysfunction can theoretically contribute to the clinical variability in PMS since six mitochondrial genes are adjacent to *SHANK3* in the 22q13.3 region [102]. These genes include cytochrome c oxidase assembly (*SCO2*) involved in the assembly of the cytochrome c oxidase; NADH dehydrogenase 1 alpha subcomplex subunit 6 *(NDUFA6*), which is necessary for the assembly of complex I of the respiratory chain; thymidine phosphorylase (*TYMP*), and tRNA 5-methylaminomethyl-2-thiouridylate methyltransferase (*TRMU*), both involved in mitochondrial DNA; carnitine palmitoyltransferase 1B (*CPT1B*), essential for fatty acid metabolism and aconitase 2 (*ACO2*), that participates in tricarboxylic acid cycle (TCA) function. In the study published by Frye et al., the activities of mitochondrial complexes I and IV were impaired when measured in the saliva of 51 individuals diagnosed with PMS [102]. Although the connection between mitochondrial dysfunction and the decline in complexes I and IV activity matches the function of the mitochondrial genes found in chromosome 22q13, little is known about the biological mechanisms between SHANKs and mitochondrial function at the excitatory synapse.

### ASD associated with mutations and loss of function of fragile X mental retardation protein

Other ASD-related proteins are not primarily associated with synaptic scaffolding but play a crucial role in synaptic signaling processes. One example is fragile X mental retardation protein (FMRP) encoded by the *FMR1* gene, which, when deleted, increases mGluR signaling [103, 104]. Expansions of a CGG repeat in the *FMR1* gene exceeding 200 bp produce a full mutation that causes the fragile X syndrome (FXS), characterized by autistic-behaviors and intellectual disability [105]. Individuals that carry the trinucleotide CGG repeat expanded between 55 and 200 bp are called premutation carriers; they do not develop FXS, but due to gene instability, the premutation expands to the full mutation, affecting the offspring [106]. Regarding metabolic function, one pioneer study showed that the brains of *Fmr1*-knockout (KO) mice had increased levels of reactive oxygen species (ROS), alterations in the expression of glutathione (GSH) in whole brains, and increased production of NADPH oxidase in specific brain regions like the cerebellum, hippocampus, and prefrontal cortex (PFC) when compared to wild-type (WT) mice [107]. Primary hippocampal neurons obtained from *Fmr1*-knockin (KI) premutation mice showed a decreased population of mitochondria and reduced mitochondrial trafficking [108]. Another study performed in primary neurons from *Fmr1*-KO mice found reduced expression of Opa1, Mfn1, and Mfn2, together with more mitochondrial fragmentation [109]. The mitochondrial fusion phenotype was rescued by increasing the expression of endogenous Huntingtin (Htt), the protein implicated in Huntington’s disease. Interestingly, Htt-KO mice display behavioral features similar to *Fmr1*-KO mice, and treatment with M1, a phenylhydrazone that permeates the cell membrane, enhances mitochondrial fusion, and restores the behavioral deficiencies in mice lacking either Fmr1 or Htt.

Mitochondrial-enriched fractions were obtained from the cortex, the hippocampus, and the cerebellum of a premutation *Fmr1*-KI mouse model. Respiration deficits were found in the hippocampus of very young animals (21 days old), characterized by lower ATP production and lower expression levels of the ATPase β-subunit (ATPB) [110]. This study emphasizes that the mitochondrial pools analyzed were non-synaptic. Here, the authors found altered gene expression of the Zn^2+^ transporters ZnT4 and ZnT6 in the mammary glands of *Fmr1*-KI dams producing reduced concentrations of Zn^2+^ in breast milk. They propose that adequate concentrations of dietary Zn^2+^ are necessary to restore normal brain metabolism [111]. Disturbances in Zn^2+^ metabolism were found in the fibroblasts from premutation individuals, possibly because of the expanded repeats that, upon accumulation, modify miRNAs processing [112].

Impaired mitochondrial energy metabolism was also reported in a study performed in the cortex of juvenile (21 days old) and adult (1-year-old) *Fmr1-*KO mice. Increased activities of the OXPHOS complexes were detected spectrophotometrically in mitochondrial membranes isolated from the cortex, but a lower ATP production was found. Similar observations were made in the striatum and cerebellum. No enhancement in the activities of the enzymes of the glycolytic pathway was present [113]. Isolated mitochondria from the forebrain of *Fmr1*-KO mice exhibited decreased mitochondrial respiration driven by complexes I and II. Reduced expression levels of ubiquinone quantified by HPLC and spectrophotometry were also detected. This study identified an augmented proton leak accompanied by reduced mitochondrial Ca^2+^ uptake that could be reversed by treatment with ubiquinone analogs in vivo and in vitro [114]. It is, therefore, not clear how Fmr1 deficiency promotes the uncoupling of respiration in this model.

A recently published study identified the translation of mitochondrial proteins in isolated synaptosomes from whole brains [115]. Kuzniewska et al. found newly synthesized mitochondrial proteins using in vitro stimulation of mouse synapses and mass spectrometry. They also analyzed the morphology and function of the synaptic mitochondria from *Fmr1*-KO mice and found more dysmorphic mitochondria and increased oxygen consumption than WT mice when measured by high-resolution respirometry.

To summarize, there is accumulating evidence showing mitochondrial dysfunction in different *Fmr1* mutant models, including Drosophila [116]. Although some of the findings point to an indirect relationship, others suggest that altered translation of mitochondrial proteins at the synapse is a possible mechanism behind disorders like FXS. More studies are necessary to understand how mitochondria contribute to the development of the FXS or if impairments in mitochondrial function are just an epiphenomenon.

## ASD-related neurodevelopmental disorders

### DiGeorge syndrome

The DiGeorge syndrome, also known as velocardiofacial or 22q11.2 deletion syndrome (22q11.2DS), is the consequence of a hemizygous microdeletion (1.5–3 Mb) on chromosome 22, with an incidence of 1 in 4000 [117]. The syndrome often presents with different neuropsychiatric symptoms [118], including schizophrenia [119]. Additionally, individuals with 22q11.2DS manifest with attention-deficit hyperactive disorder (ADHD), ASD, anxiety, depressive, and bipolar disorders [120, 121].

Recent research in a hemizygous mouse model with a 1.5-Mb deletion in chromosome 22q11 (LgDel 22q11) demonstrates that stabilization and enhancement of mitochondrial function by the administration of N-acetyl cysteine constitute a viable therapeutic approach to treat cortical under-connectivity [122], a frequent pathogenic feature in neurodevelopmental disorders. Mitochondria are affected in this study, specifically in cortical layer 2/3, with morphological impairments characterized by the absence of cristae and increased ROS production in the mitochondria and the cytosol. This study highlights the relevance of mitochondria in synapses and their implications in normal and neuronal disease states and emphasizes existing differences in the mitochondrial population depending on brain regions. Others reported alterations in the mitochondrial proteome of the hippocampus and prefrontal cortex in a 22q11.2 microdeletion mouse model [123]. Specifically, the mitochondrial transporters encoded by *Slc25a1*-*Slc25a4* appear to play a significant role in synapse formation. Likewise, reduced expression of another gene located on 22q11.2, the gene encoding the mitochondrial large ribosomal subunit protein 40 (*Mrpl40*), impairs short-term potentiation (STP) and mitochondrial Ca^2+^ buffering properties in axons of the CA3 hippocampal region [124]. Although no mitochondrial morphology changes were detected, overexpression of the mitochondrial adenine nucleotide translocase protein (Ant1) encoded by the *Slc25a4* gene recovered the STP to WT levels, supporting the role of mitochondrial transporters in this microdeletion syndrome.

### Rett syndrome

Rett syndrome (RS) is the result of mutations in the X-linked gene methyl-CpG-binding protein 2 (*MECP2*). Patients with RS have impairments in language and communication, learning and coordination and they show autistic-like behaviors [125, 126]. The syndrome also presents with motor difficulties and microcephaly and patients grow slower than other children [127]. More elongated mitochondria were identified in the dendrites and axons from the hippocampus of a mouse model lacking exons three and four of the *Mecp2* gene (*Mecp2B*) compared to WT [128]. The hippocampus from male *Mecp2* mutant mice also displays an increased expression of superoxide dismutase 1 (SOD1) and downregulation of the Ca^2+^ channel Cacna1g, resulting in augmented oxidative stress as detected by optical recordings obtained with the redox probe roGFP1—that measures both mitochondrial and cytosolic ROS content—in hippocampal slices [129]. Oxidative damage was also detected in whole brains of *Mecp2*-KO and *Mecp2-308* mutant mice [130]. The treatment with free radical scavengers like Trolox—a derivative from vitamin E—rescued mitochondrial functionality and restored synaptic LTP in the hippocampus and hippocampal astrocytes of this RS mouse model [131, 132]. Other compounds such as metformin and quercetin rescued the mitochondrial phenotype in whole brains of *Mecp2-308* mutant mice and primary astrocytic cultures, respectively [133, 134]. The downregulation of Mecp2 in microglia generates increased oxygen consumption, less ATP production, and overexpression of Snat1—a glutamine transporter encoded by *Slc38a1*, a Mecp2 target gene previously identified in a ChIP-seq assay [135]. Mecp2 intervenes in regulating the expression of *Slc25a4 *encoding Ant1 [136]. Forlani et al. found increased mRNA expression of *Slc25a4* in whole brains and the cerebellum of *Mecp2*-KO mice. Ant1 has been associated with mitochondrial disorders like Senger’s syndrome, in which skeletal muscle mitochondria are impaired [137, 138]. Another mitochondrial gene found overexpressed in the cortex and cerebellum of *Mecp2*-KO mice is *Prodh*, which encodes the proline oxidase protein localized in the inner-mitochondrial membrane [139]. Mutations in proline oxidase are associated with behavioral deficits, epilepsy, and intellectual disabilities [140].

Furthermore, it was shown that the brains of *Mecp2-308* mutant females produce more hydrogen peroxide (H_2_O_2_) and exhibit a decreased mitochondrial membrane potential (Ψ_m)_ and diminished mitochondrial ATP production [141]. This study also found reduced activities of the respiratory chain complexes in the hippocampus and cortex, measured by spectrophotometric assays. Complexes I, II, and V were specifically less active in the cerebellum and striatum. The treatment of these mice with cytotoxic necrotizing factor 1 (CNF1), a bacterial protein previously used for ameliorating the behavioral phenotype in the same mouse model, recovers the activities of the respiratory complexes to WT levels [142]. It was also reported that mitochondrial dysfunction could be recovered after treatment with the selective agonist LP-211, which stimulates the brain serotonin receptor 7 (5-HT7R). However, no underlying mechanisms about the role of serotonin and mitochondria in this syndrome have been established [143].

A study performed in isolated mitochondria from the hippocampus and cortex of *Mecp2*-KO mice indicated that hippocampal and cortical mitochondria are impaired as they release more ROS and consumed more O_2_ compared to WT littermates [144]. Still, it is unclear if the mitochondrial defects found in these disease models can be solely attributed to increased respiration and if these alterations are the consequence of redox imbalances. Similar results were obtained in a previous study performed in isolated mitochondria from whole brains of *Mecp2*-KO mice that also found significantly higher respiration rates than WT, and the mitochondrial ubiquinol-cytochrome c reductase core protein 1 (*Uqcrc1*) gene was upregulated [145]. To summarize, there is considerable evidence linking RS and mitochondrial dysfunction in patients and animal models of the disease [146]. Interventions targeting mitochondria might be beneficial in the treatment of RS.

### Tuberous sclerosis complex

Tuberous sclerosis complex (TSC) is a rare disease that affects several organs and the central nervous system. It is estimated that 61% of patients with this syndrome are affected by ASD [147]. TSC is caused by mutations in the *TSC1* and *TSC2* genes, which modulate the mammalian target of rapamycin complex 1 (mTORC), a metabolic sensor [148]. Hippocampal and cortical neurons with a decreased expression of Tsc have less functional mitochondria in the axons, a decline in basal respiration, ATP turnover, total respiratory capacity, and Ψ_m_, together with mitophagy defects [149]. Similar mitochondrial morphological deficits were found in callosal projection axons in a mouse model of the conditional expression of Tsc1 with synapsin I promoter–driven Cre recombinase allele (Tsc1cc; Syn1-Cre+) [150], which recover a WT-like morphology after treatment with rapamycin [151].

### Syndromic ASD caused by 15q11-q13 deletions, microdeletions, and duplications

The gene encoding ubiquitin-protein ligase E3A (*UBE3A*) is localized to the 15q11-15q13 chromosome in humans. UBE3A deficiency is responsible for Angelman syndrome (AS), a severe neurological condition characterized by cognitive impairment and developmental delay. Patients with AS often present with ASD features, although there is an unresolved controversy about considering AS and ASD independent from each other [152]. Increased expression of the *Ube3a* gene in mice produced autistic-like behaviors [153]. Additionally, reduced function of complex III in patients with the 15q11-q13 duplication syndrome has been documented [154]. The role of mitochondria has been studied mainly in AS mouse models, like the heterozygous mice *Ube3a*^*m-\p+*^ [155], in which Ube3a was found in close proximity to the outer mitochondrial membrane [156]. Mitochondrial morphological changes in the CA1 hippocampal region were found, like smaller mitochondria and disturbances in mitochondrial cristae compared to WT littermates [157]. Mitochondria were isolated from the hippocampus, cortex, and cerebellum, and the activities of the different components of the electron transport chain (ETC) were tested [158]. A significantly reduced activity of complex III was found in the cortex and hippocampus. Electron flow was recovered by administering a Coenzyme Q10 (CoQ10) analog, increasing the protein expression levels of complexes III and IV in neurons from hippocampal regions CA1, CA2, and CA3 and the cerebellum. Nonetheless, oxidative stress was decreased only in the hippocampus when measuring the GSH and glutathione disulfide (GSSG) ratio, indicating that this CoQ10 analog influences mainly bioenergetics, and other interventions are necessary to counteract the effects of oxidative stress. In the same AS mouse model, the mitochondrial-targeted antioxidant MitoQ 10-methanesulfonate (MitoQ), which traverses the blood-brain barrier and accumulates in mitochondria [159], showed promising results by reducing ROS levels in the CA1 hippocampal region and improving synaptic plasticity [160]. A study performed in a mouse model of Prader-Willi syndrome—another neurodevelopmental disorder that displays variable symptomatology and includes ASD in some individuals [161]—found differential expression of 66 mitochondrial genes including those encoding ribosomal proteins and the mitochondrial transporter Aralar1 in whole brains [162].

### Genetic mutations related to an ASD-like clinical syndrome

Down syndrome (DS) or trisomy 21 represents a genetic form of mental retardation caused by the overexpression of genes located on chromosome 21, with a high incidence of Alzheimer’s disease (AD) [163]. The prevalence of ASD in DS appears to be between 1 and 11% [164]. The rates vary considerably depending on the sample size and diagnostic criteria employed in each study [165–169]. Additionally, ASD diagnosis is challenging in the DS population due to the intrinsic behavioral and language impairments associated with this syndrome [170]. DS and mitochondrial dysfunction have been assessed in different models, including fibroblasts derived from patients, neuronal cultures derived from fetal tissue, and post-mortem brain tissue of DS individuals [171, 172]. One study explored the role of mitochondria in isolated neural progenitor cells obtained from the dentate gyrus of the well-characterized DS mouse model *Ts65Dn* [173]. The mitochondria from these cells were more fragmented, showed reduced ATP production, and increased expression of the fission protein Drp1. The mitochondrial phenotype of these cells was reversed by treatment with mitochondrial division inhibitor 1 (Mdivi-1). Mitochondrial functional impairment was also identified in cultured hippocampal neurons and astrocytes from the *Ts1Cje* DS mouse model, showing reduced Ψ_m_, decreased ATP levels, and augmented oxidative stress [174]. Antioxidants like epigallocatechin-3-gallate (EGCG) and vitamin E were tested in mouse models of DS [175–177], showing recovered LTP in hippocampal slices and increased neuronal populations in the dentate gyrus of *Ts65Dn* mice, respectively.

Another ASD-associated gene related to mitochondrial function, specifically to mitophagy, is the WD repeat and FYVE domain-containing 3 (*WDFY3*) [178]. A study published by Napoli et al. used *Wdfy3*^*+/lacZ*^ mice and obtained mitochondrial-enriched fractions of the whole brain for proteomic analysis [179]. They found altered mitochondrial function in the cortex, cerebellum, and dendrites of primary cortical neurons derived from these mice. Abnormal morphology, budding protrusions, and a significant number of round mitochondria were present. This study also suggested that Wdfy3 participates in mitochondrial transport and mitophagy. The accumulation of defective mitochondria affects fatty acid β-oxidation (FAO) and, eventually, neuronal differentiation since FAO controls the shift from neural stem cells to intermediate progenitor cells during brain development in mice [180].

Mitochondrial abnormalities were identified in the inbred BTBR+tf/j (BTBR) mouse line. Isolated mitochondria from the BTBR brain at postnatal day 35 consume less oxygen at basal conditions than controls. Complex II activity was reduced, and a fragmented mitochondrial shape was found in neocortical tissue. The mitochondrial phenotype in this model could be reversed by administering a ketogenic diet (KD) [181]. BTBR mice fed with KD for 14 days showed discrete changes in the mRNA expression of mitochondrial bioenergetic transcripts in the hippocampus and temporal cortex [182]. Another study performed with the BTBR mouse model found that a KD affects mitochondrial function mainly in the liver, and although an increased gene expression of *Fis1*, *Drp1*, *Mff*, *Opa1*, and the gene encoding the BCL2/adenovirus E1B 19kd-interacting protein 3 (*Bnip3*) in the PFC was found, no significant alterations in protein expression were detected [183].

Mitochondrial respiration using succinate as substrate and the maximal oxygen capacity evaluated after the addition of the mitochondrial uncoupler carbonyl cyanide-p-trifluoromethoxyphenylhydrazone (FCCP) was reduced in isolated mitochondria from the hippocampus of BTBR compared to WT mice [184]. Normal levels of succinate-driven respiration were restored by treatments with anti-inflammatory palmitoylethanolamide (PEA). Decreased SOD activity was found in the hippocampus of BTBR mice when assessed spectrophotometrically. The SOD activity was rescued after PEA administration for 10 days.

Many of the studies performed in post-mortem brains of ASD patients provide evidence of mitochondrial abnormalities, both morphological and functional [185]. However, as in many other diseases, it is unknown whether those changes are the consequences of upstream signaling cascades, synaptic impairment, or immunological responses. Mutations in mitochondrial genes have been postulated to confer ASD vulnerability, like *SLC25A12*, which codes for the isoform 1 of the Ca^2+^ regulated mitochondrial aspartate-glutamate carrier Aralar (Aralar1) [186]. Nevertheless, ASD susceptibility conferred by the *SLC25A12* gene is only present in specific populations.

Another gene of the solute carrier family associated with autism is *SLC7A11* [187]. The *SLC7A11* gene codes for xCT, a subunit of the astrocytic cystine-glutamate antiporter system (xc-). This transport system is necessary for redox balance and regulation of extracellular glutamate concentrations [188] by cooperating with excitatory glutamate transporters [189]. The deletion of xCT in mice produced less presynaptic mitochondria in the dorsolateral striatum and behavioral deficits associated with ASD [190]. This study highlights the significant role of astrocytes in ASD. The mitochondrial proteome composition differs according to brain cell type, showing differences in beta-oxidation between neurons and astrocytes [191].

**Fig. 3:**
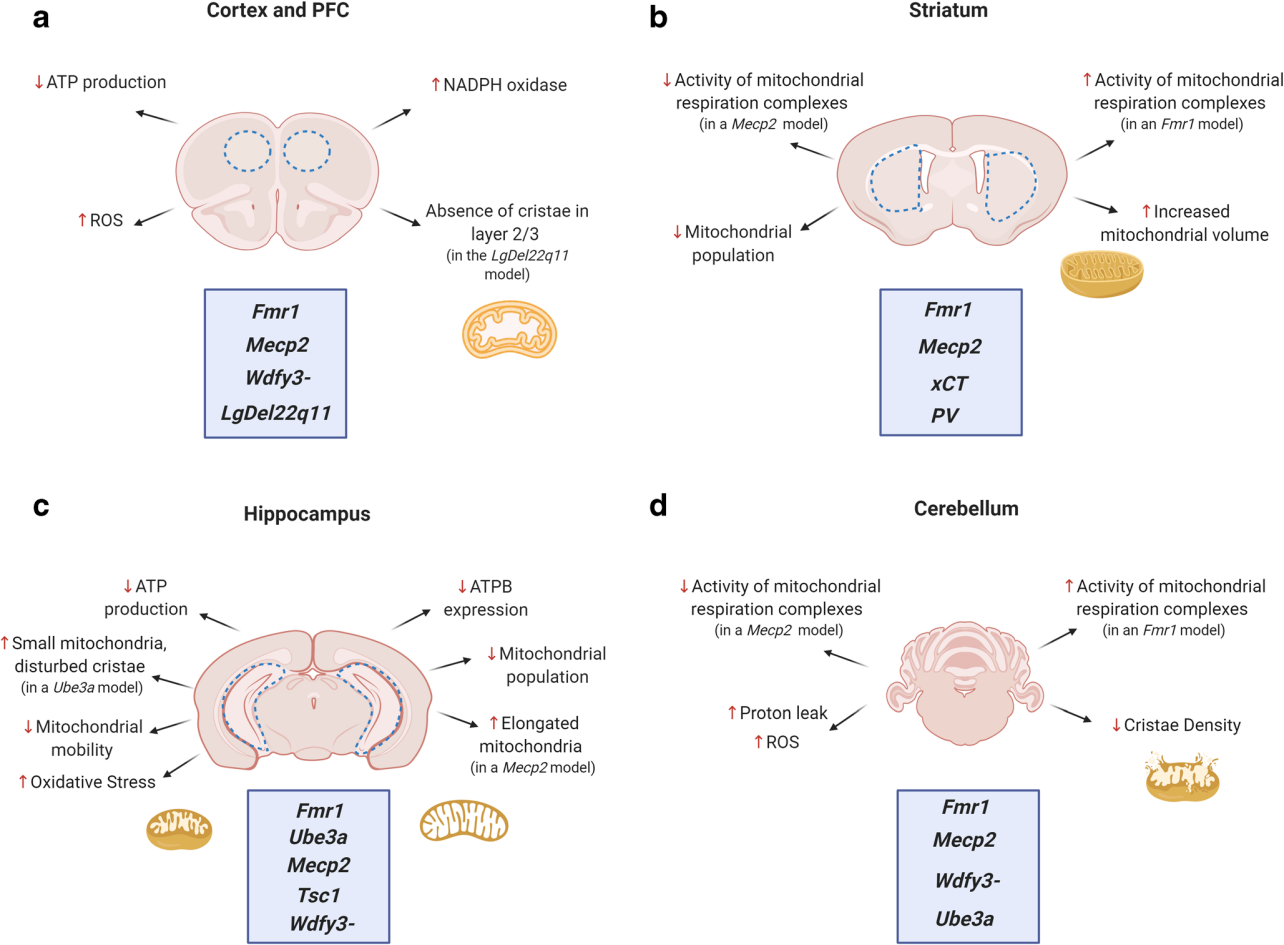
Mitochondrial disturbances in different brain regions of mouse models of ASD. The figure provides an overview of the mitochondrial defects found in the cortex/prefrontal cortex (PFC), striatum, hippocampus, and cerebellum in different ASD mouse models. Arrows point to the specific mitochondrial disturbances found in the different mouse models in **a** cortex and PFC; **b** striatum; **c** hippocampus and **d** cerebellum. Dotted blue lines delineate the specific brain regions. Most of the studies are focused on hippocampal mitochondria; the mitochondrial phenotype has been most extensively studied in *Fmr1* and *Mecp2* mutant mouse models. Created with BioRender.com

GABAergic neurons that contain parvalbumin (PV+) are of particular interest in ASD [192, 193]. PV binds Ca^2+^ through EF-hand domains and participates in buffering intracellular [Ca^2+^] [194–196]. The absence of PV in mice recapitulates ASD features, and a recent study showed that mitochondrial morphology is affected in this model, showing increased density and volume in the soma of neurons from the striatum, the medial PFC, the somatosensory cortex, the thalamic reticular nucleus, Purkinje neurons, and molecular layer interneurons [197]. Table 1 and Fig. 3 summarize the mitochondrial defects found in different brain regions and the models involved.

**Table 1:** Mitochondrial disturbances found in specific brain regions of ASD and ASD-related neurodevelopmental disease mouse models

Author	Mitochondrial disturbances	Model	Brain regions evaluated
Bekay et. al 2007 [107]	Higher levels of ROS, NADPH oxidase activation, altered expression levels of the GSH system	*Fmr1*-KO mice	Hippocampus, PFC, cortex, cerebellum
Kaplan et. al 2012 [108]	Decreased number, mobility, and metabolic function	Premutation *Fmr1*-KI mice	Primary hippocampal neurons
Napoli et. al 2016 [110]	Lower ATP production and lower expression of ATPB	Premutation *Fmr1*-KI mice	Hippocampus
D’Antoni et. al 2019 [113]	Hyperactivation of mitochondrial respiration complexes, lower ATP production in cortex	*Fmr1*-KO mice	Cortex, striatum, cerebellum
Shen et. al 2019 [109]	Reduced expression of mitochondrial fusion proteins, more fragmented mitochondria	*Fmr1*-KO mice	Primary hippocampal neurons
Griffiths et. al 2020 [114]	Decreased respiration, augmented proton leak, reduced mitochondrial Ca^2+^ uptake	*Fmr1*-KO mice	Isolated mitochondria from forebrain
Kuzniewska et. al 2020 [115]	Abnormal mitochondrial morphology, increased respiration	*Fmr1*-KO mice	Synaptic mitochondria
Devaraju et. al 2017 [121]	Impaired STP and mitochondrial buffering in the synapse	*Mrpl40*^*+/-*^ mice	CA3 hippocampal region
Fernandez et. al 2019 [122]	Absence of cristae, increased ROS production	*LgDel 22q11* mice	Cortex (layer 2/3)
Gokhale et. al 2019 [123]	Mitochondrial proteome affected	*Df(16)A*^*+/-*^ mice	Hippocampus, PFC
Kriaucionis et. al 2006 [145]	Increased respiration	*Mecp2*-KO mice	Mitochondria isolated from whole brains
Urdinguio et. al 2008 [139]	Increased expression of *Prodh*	*Mecp2*-KO mice	Cortex, cerebellum
Belichenko et. al 2009 [128]	Increased elongated mitochondria	*Mecp2B* mice	Hippocampus
Forlani et. al 2010 [136]	Increased expression of *Slc25a4*	*Mecp2*-KO mice	Whole brain, cerebellum
Grosser et. al 2012 [129]	Increased SOD1 expression, increased ROS production	*Mecp2-308* mice	Hippocampus
DeFelice et. al 2014 [130]	Increased oxidative stress	*Mecp2*-KO and *Mecp2-308* mice	Whole brain
Janc et. al 2014 [131]	Decreased FAD/NADH ratio, reduced Ψ_m_	*Mecp2*-KO mice	CA1 hippocampal region
Jin et. al 2015 [135]	Increased oxygen consumption, reduced ATP production, overexpression of Snat1	*Mecp2*-KO mice	Microglia
DeFilippis et.al, 2015 [141]	Reduced activity of complexes II, III, IV, and V	*Mecp2-308* mice	Mitochondria isolated from hippocampus and cortex
DeFilippis et.al, 2015 [142]	Increased ROS and diminished ATP production in mitochondria isolated from whole brain and cerebellum, Reduced activity of complexes I, II, and V in mitochondria isolated from striatum and cerebellum	*Mecp2-308* mice	Mitochondria from whole brain, striatum, and cerebellum
Bebensee et. al 2017 [132]	Augmented mitochondrial number, increased oxidative stress	*Mecp2*-KO mice	Primary hippocampal astrocytes
Valenti et. al 2017 [143]	Reduced ATP production and ATP concentration, reduced activities of ETC complexes, increased ROS production	*Mecp2-308* mice	Mitochondria isolated from whole brain
Can et. al 2019 [144]	Increased ROS, intensified oxygen consumption	*Mecp2*-KO mice	Hippocampus, cortex
Zuliani et. al 2020 [133]	Reduced ATP production, reduced activities of mitochondrial complexes II and V, decreased protein expression of ETC complexes, increased oxidative stress	*Mecp2-308* mice	Whole brain
Dave et. al 2020 [134]	Reduced activities of ETC complexes, reduced Ψ_m_	*Mecp2*-KO astrocytes	Primary cultured astrocytes
Ebrahimi-Fakhari et. al 2016 [149]	Accumulation of mitochondria in the cell bodies, decreased Ψ_m,_ decreased baseline respiration, ATP and total respiratory capacity	*Tsc1cc*; *Syn1-Cre +* mice, iPSC-derived cortical neurons	Hippocampal and cortical neurons, iPSC-derived cortical neurons from TSC patients
Su et. al 2011 [157]	Impaired morphology, decreased activity of complex III	*Ube3am-\p+* mice	CA1 hippocampal regions, hippocampus
Yazdi et. al 2013 [162]	Differential expression of mitochondrial genes	*PWS-IC del* mice	Whole brain
Santini et. al 2015 [160]	Enhanced levels of superoxide	*Ube3am-\p+* mice	Hippocampal and cerebellar acute slices Hippocampus
Llewellyn et. al 2015 [158]	Reduced activity of complex III, increased oxidative stress	*Ube3am-\p+* mice	Cortex, hippocampus
Shukkur et. al 2006 [174]	Decreased Ψ_m_, decreased ATP production, increased oxidative stress	*Ts1Cje mice*	Hippocampal neurons Dentate Gyrus
Valenti et. al 2017 [173]	Fragmented morphology, decreased ATP production, increased expression of Drp1	*Ts65Dn mice*	Dentate Gyrus
Napoli et. al 2018 [179]	Lower ATP production, decreased cristae density, reduced OXPHOS capacity, increased ROS, increased proton leak	*Wdfy3-* haploinsufficient mice	Cortex, hippocampus, cerebellum (most affected)
Newell et. al 2016 [183]	Increased expression of genes associated with mitochondrial fission/fusion after administration of KD Increased expression of Bnip3	BTBR mice	PFC
Mychasiuk et. al 2017 [182]	Changes in expression of mitochondrial bioenergetics genes after administration of KD	BTBR mice	Isolated mitochondria from hippocampus and cortex
Cristiano et. al 2018 [184]	Reduced oxygen consumption, reduced SOD activity, increased ROS production	BTBR mice	Isolated mitochondria from hippocampus, hippocampus
Ahn et. al 2020 [181]	Reduced oxygen consumption, round shape mitochondria, increased expression of phosho-Drp1 at Ser616 and phospho-Mff at Ser146	BTBR mice	Isolated mitochondria from Neocortex, primary cortical neurons
Bentea et. al 2020 [190]	Reduced presynaptic mitochondrial population	*xCT*^*-/-*^ mice	Striatum
Janickova et. al 2020 [197]	Increased mitochondrial volume/density	PV-deficient mice	Neurons from the striatum, the medial PFC, the somatosensory cortex, the thalamic reticular nucleus, Purkinje neurons, molecular layer interneurons

## Concluding remarks

The complexity of ASD is characterized by mitochondrial dysfunction, among other factors. Evidence for the involvement of mitochondria in ASD is increasing and has been tested in multiple models [198–202]. Mitochondrial disease also co-occurs with ASD and is sometimes difficult to distinguish [203]. However, no precise biological mechanisms are available to explain mitochondrial dysfunction and ASD, and studies directed to evaluate the efficacy of mitochondrial-targeted compounds in patients are scarce [204].

Currently, it is widely accepted that mitochondrial function varies in different parts of the brain [205, 206] and their role at the synapse goes beyond providing energy and stabilizing Ca^2+^ concentrations [207]. Studies focusing on mitochondria at the synapse are necessary to distinguish their role between the pre- and postsynapse in ASD and ASD-related neurodevelopmental disorders. Although most of the studies have focused on hippocampal mitochondria, the cerebellum and the cortex have a distinctive mitochondrial phenotype that can contribute to the variability of the symptoms observed in patients. Other mitochondrial populations need to be studied, like the ones from the amygdala and striatum, brain regions that participate in the conceptualization and execution of emotions, memory, learning habits, and motor skills [208, 209]. For example, the striatum has a higher OXPHOS activity than the hippocampus and cortex and is especially sensitive to Ca^2+^ overload [210, 211]. Understanding how synaptic mitochondria contribute to different neuronal populations in disease states could help to elucidate and develop novel therapeutic options to treat ASD and ASD-related neurodevelopmental disorders more effectively.
